# Advances in Sensing Technologies for Monitoring of Bone Health

**DOI:** 10.3390/bios10040042

**Published:** 2020-04-21

**Authors:** Seema Rani, Sanchita Bandyopadhyay-Ghosh, Subrata Bandhu Ghosh, Guozhen Liu

**Affiliations:** 1 Engineered Biomedical Materials Research and Innovation Centre (EnBioMatRIC), School of Automobile, Mechanical and Mechatronics Engineering (SAMM), Manipal University Jaipur, Rajasthan 303007, India; 2 Graduate School of Biomedical Engineering, Faculty of Engineering, The University of New South Wales, Sydney NSW 2052, Australia

**Keywords:** bone, bone remodeling, bone biomarkers, bone diseases, osteoporosis, biosensors

## Abstract

Changing lifestyle and food habits are responsible for health problems, especially those related to bone in an aging population. Poor bone health has now become a serious matter of concern for many of us. In order to avoid serious consequences, the early prediction of symptoms and diagnosis of bone diseases have become the need of the hour. From this inspiration, the evolution of different bone health monitoring techniques and measurement methods practiced by researchers and healthcare companies has been discussed. This paper focuses on various types of bone diseases along with the modeling and remodeling phenomena of bones. The evolution of various diagnosis tests for bone health monitoring has been also discussed. Various types of bone turnover markers, their assessment techniques, and recent developments for the monitoring of biochemical markers to diagnose the bone conditions are highlighted. Then, the paper focuses on the potential assessment of the recent sensing techniques (physical sensors and biosensors) that are currently available for bone health monitoring. Considering the importance of electrochemical biosensors in terms of high sensitivity and reliability, specific attention has been given to the recent development of electrochemical biosensors and significance in real-time monitoring of bone health.

## 1. Introduction

Growing and living bone tissue forms part of the vertebrate skeleton. Bone is basically a combination of organic matrix, inorganic minerals (calcium phosphate), and vitamins that makes the structural framework. Type I collagen forms approximately 94% of the organic bone matrix. During development of skeleton, modeling and remodeling of bone occur simultaneously [1]. Bone modeling is a slow and continuous formation of bones by connective tissues until the age of adolescence, as bones are not fully developed at the time of birth. Bone remodeling is also a continuous process by which mature bone tissues are removed and replaced with newly synthesized bone. This process is also known as bone turnover. Osteogenic cells, osteoblasts, osteoclasts, and osteocytes are four different type of bone cells involved in progression of bone modeling and remodeling [2]. Osteoblasts are responsible for bone formation; osteoclasts enable the bone resorption. Bone lining cells cover bone surfaces that take minerals directly and release them in bone and osteocytes behave as natural mechanosensors [3]. During osteoblastic bone formation, procollagen I aminioterminal propeptide (PINP) and osteocalcin (non-collagenous protein) are either found in the cavities of bone matrix or in the blood circulation [4,5]. In osteoclastic resorption, collagen is degraded, and small peptide fragments are released in the blood. In addition, bone resorption markers such as cross-linked N-terminal and C-terminal telopeptides of type I collagen are released in urine. The degraded collagen and peptides behave as biochemical markers [6]. The identification of bone biomarkers is important in the timely diagnosis of diseases such as osteoporosis, bone cancer, and infections, with their underlying processes involved. Biomarkers for bone health can be specific cells, enzymes or hormones, and gene products. The accurate recognition and appearance of specific bone biomarkers can be supportive in staging the diagnosis and effective treatment of bone diseases [7]. An electronic device is needed to process this biological information into readable output. However, it is quite challenging to connect such a device to a biological environment due to the complexity of attaching the device and processing the electronic signals. Besides, such devices are costly, require expertise, and can detect bone health only after large amounts of degradation of bone.

**Figure 1:**
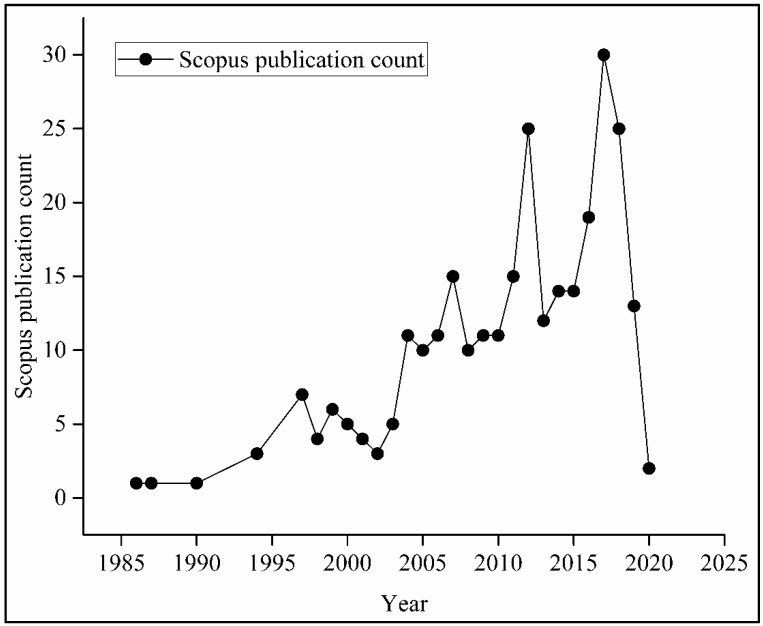
Research documents published on bone biosensors by year (Scopus data up to 15 January 2020).

In this regard, several studies have reported that more sensitive and real-time assessment tools are required. Research has been continuously going on in the field of biosensors for the assessment of bone health by using biochemical markers present in biological samples such as blood or urine [8]. Biosensors collect information from biofluid and convert it into an electronic signal. In recent years, literature regarding biosensor technology has shown its potential as a tool for the prognostic monitoring of abnormal changes in bone mineral density. The development of bone biosensors is one of the rapidly emerging fields of biosensors. Researchers started exploring this area in the mid-1980s and only after 2005 did work on bone biosensors started gearing up. Later on, a significant rise in publication can be observed (Figure 1), demonstrating the increasing need and importance of working on bone biosensors.

Against the above background, this paper reviews and provides a potential assessment of recent bone biosensing techniques based on biochemical marker-based sensors. Biosensors for the detection of biomarkers to indicate bone health are increasingly becoming popular, since biomarkers are readily available in serum or urine carrying bone health information. In the following section, various methods for diagnosis based on biochemical changes associated with bone formation and resorption along with the analytical techniques for their measurements have also been reported. Finally, reports of traditional and emerging technologies on the development of biosensors for the assessment of bone health have been discussed.

## 2. Bone Remodeling

**Figure 2:**
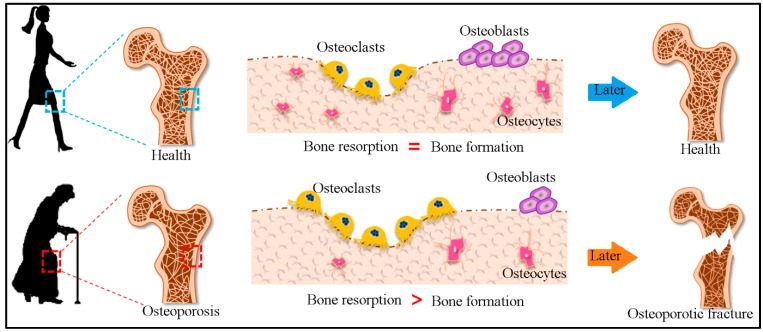
The process of bone remodeling, resulting from continuous bone resorption and bone formation with increasing age, and the balance shifts to more bone resorption than bone formation, causing osteoporosis (reproduced with permission from reference [9]).

Bone remodeling is a lifelong process of new bone formation and the resorption of old bones. Osteoblasts synthesize and release a range of proteins such as extracellular matrix proteins, collagen, cytokines, and growth factors responsible for bone formation and changes in the extracellular matrix into bone through mineralization [9]. The components available prior to the formation of the organic matrix are called osteoids, and their mineralization is dependent on Ca^2+^ available in plasma [10]. Simultaneously, osteoclast cells accountable for resorption of bone are also found on the small depressions of bone surface. A variety of enzymes produced by osteoclasts dissolve the calcium of bone and the bone matrix. Mineralized bone is broken down, and collagen threads are absorbed by osteoclasts. Bone remodeling is controlled through the interplay between osteoblast differentiation and osteoclast activation and varies with age, as shown in Figure 2 [11]. Bone turnover markers (BTM) give real-time information about the bone remodeling process. It is useful for the monitoring of bone health [12].

## 3. Bone Diseases

The skeleton stores calcium and minerals for its proper operation. These calcium and minerals are not synthesized by body but taken from food. If enough calcium is not taken in the diet, the stored calcium of bone is utilized for the proper functioning of the body, making bones weaker [13,14,15,16]. Metals and minerals such as phosphorus, calcium, magnesium, sodium, potassium, sulfur, iodine, iron, molybdenum, manganese, and zinc are very important for human health. However, little deficiency or an excess of them can upset a delicate health balance of body. In addition, they are considered infamous for encouraging many chronic situations, including carcinogenesis [17,18]. Calcium is the most important mineral for bone strength and health, as a deficiency of calcium may cause various diseases. The body absorbs calcium from food with the help of vitamin D to build bones and teeth; a poor lifelong intake of calcium and vitamin D causes weaker bone growth and leads to disorders [19].

There are various types of bone problems such as osteoporosis, low bone density, osteogenesis imperfecta, and Paget’s disease. These diseases of bone make them weak [20]. The World Health Organization (WHO) has reported various bone densities associated with normal and diseased bone [21] and categorized them on the basis of T-score range where the standard deviation (SD) of bone mineral density (BMD) of young adult reference mean indicates bone health. Adding to the above, increasing the metal intoxication and contaminated environment due to human activities has brought significantly adverse effects on health. Toxic metals such as lead can enter into the body, get deposited in different organs including bone, and cause serious damages [22]. Similarly, low concentrations of aluminium, zinc, chromium, arsenic, copper, nickel, and mercury are also found to be toxic for bone cells and may get accumulated in the bone matrix, causing various diseases [23].

The following section covers different type of bone diseases, their underlying mechanisms, and associated problems:

### 3.1. Osteoporosis

**Figure 3:**
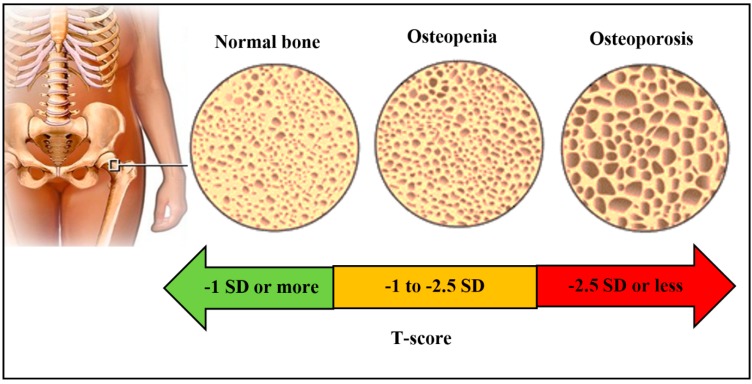
Comparative view of normal bone, osteopenia, and osteoporosis [26] (Reproduced under the terms of the ‘Creative Commons Attribution’ for Open Access content).

In this silent disease, bone becomes fragile and porous, and it may easily break (Figure 3). During growing age, bone formation is faster than resorption, which makes bone heavier, denser, and stronger. Balance in this process enables proper bone growth, failing which may cause a lowering of bone mass and structural weakening of bone tissues, which leads to osteoporosis and makes them prone to fracture at the wrist, spine, and hip. There are various habits of poor lifestyle such as less physical work, smoking, drinking alcohol, habits of junk food, and less intake of vitamin D that can result in osteoporosis [24,25]. Low bone density or osteopenia is a state of reaching the threshold value of BMD for osteoporosis. Osteopenia is not considered under any disease category. Similar to how prehypertension and prediabetes are early signs of hypertension and diabetes, osteopenia gives early indications of osteoporosis.

### 3.2. Bone Cancer and Infections

Several infections and cancer also occur in bone because of unbalanced osteoclast activities, either systemically as in humoral hypercalcemia of malignancy (HHM) or regionally in bone metastasis [27]. Bone metastasis occurs when cancer cells relocate in fresh bone. Parathyroid hormone-related protein (PTHrP) increases resorption and overrides normal calcium homeostasis, leading to such diseases [28]. Bone cancer can be divided into two categories: primary bone cancer and secondary bone cancer. If cancer starts in bone cells either at its outer surface or from the center of bone, it is called primary bone cancer, while cancer that occurs in any part of body and spreads to the bone is known as secondary bone cancer [29].

### 3.3. Osteogenesis Imperfecta

Osteogenesis imperfecta is a rare genetic disorder that affects the connective tissues of bone, making them fragile with low bone mass. It affects the ability of the body to build stronger bones. Primarily, it occurs due to alterations in the genes (COL1A1 and COL1A2) that encrypt type I procollagen [30]. The severity of the disorder may vary with different symptoms such as blue sclera, hyperlaxity of the skin and ligaments, hearing impairment, and intrauterine fractures [31]. Osteogenesis imperfecta occurs due to the deficiency of proteins which interacts with collagen and influences its post-translational alteration [32].

### 3.4. Paget’s Diseases of Bone

Paget’s disease is a metabolic bone disorder found in elderly people age 55 and above. As the age increases, chances of Paget’s diseases rise 8% in men and 5% in women above 80 years. It is a serious bone disease that occurs due to unbalanced bone remodeling [33]. Abnormal behavior during osteoclast and too much bone formation during osteoblast results in expanded and disfigured bone. Paget’s disease affects various bones such as the spine, skull, hip, tibia, and ribs. In Paget’s disease, the level of bone metabolism markers such as alkaline phosphatase is increased [34]. Paget’s disease of bone may develop cancer in bone [35].

### 3.5. Osteomalacia

Bones become softer in osteomalacia. The outer surface of bone (cortex) is made up of minerals mainly including phosphate, vitamin D, and calcium, while a softer inner matrix consists of collagen fibers [36,37]. Improper mineralization and the toxic effects of drugs make the bone softer in osteomalacia [38,39]. Other various reasons such as a deficiency of vitamin D, bypass surgery of the small intestine, celiac disease, kidney, or liver disorders, tumors, and drugs can also make the bone softer [40]. Osteomalacia is also known as rickets if it occurs in childhood [41].

### 3.6. Osteopetrosis

Osteopetrosis is a hereditary bone disorder that makes bone abnormally dense and fragile. The bone density is increased due to unbalanced resorption by osteoclast cells. Osteopetrosis are found in three different forms: osteopetrosis tarda, osteopetrosis congenita, and marble bone. Osteopetrosis tarda is a benign form diagnosed in adulthood, while osteopetrosis congenita and marble bone are malignant variants found during infancy and childhood, respectively [42].

### 3.7. Fibrous Dysplasia

Fibrous dysplasia is a bone disorder in which abnormal fibrous tissues are developed in the place of normal bone. In this, osteoblast cell fails to mature and hence produces abnormal fibrous tissue, which further grows and causes weakening or a deformation of bone. However, it does not spread from one bone to another [43]. This rare bone disorder is mostly developed in children and young adults [44]. Fibrous dysplasia can affect the skull, femur, tibia, pelvis, humerus, and ribs [45]

### 3.8. Scoliosis

This type of bone disorder results in the deformation of bone when either the spine gets curved in sideways or it takes the form of a letter “C” or “S”. It may occur in children and adolescents [46,47]. Scoliosis is a genetic disorder that may vary in intensity from mild to severe amongst individuals. There are mainly three type of scoliosis: idiopathic scoliosis is hereditary, congenital scoliosis is a rare spine abnormality detected at birth, and neuromuscular scoliosis is caused by abnormality in the muscles and nerves that support the spine [42].

### 3.9. Osteomyelitis

The swelling of bone tissue due to infection is known as osteomyelitis. It can be a bacterial infection of blood that may spread to bone [48]. It can affect children’s long bones and adult bones of the spine [49]. It may occur due to surgery, frequent medicine injections, diabetes, and a weak immune system [50,51]. Osteomyelitis is diagnosed through bone biopsy, and treatment requires extra care with multiple surgery [52].

## 4. Current Diagnostic Tests for Bone Health Monitoring

Several methods are commercially available for the diagnosis of bone health.

### 4.1. Bone Densitometry

Bone densitometry is also known as dual-energy X-ray absorptiometry (DEXA) [53]. It was developed in the 1980s with widespread use started in 1988. Today, it is a reliable, popular, and most completely developed method in use. In this method, images of the inside bone are produced by exposing the skeletal site to two X-ray beams having different intensities of ionized radiation. Using two measurement results, the mineral content of bone is calculated through a computer. It can further be used for an analysis of osteoporosis [54]. Bone mineral density (BMD) results are useful in precisely predicting fracture risks with low ionizing radiation dose. However, the bone densitometry technique detects the bone health after the occurrence of bone loss. It cannot differentiate between osteoporosis and osteomalacia. In addition, the studies may take 2–3 years of continuous monitoring to detect significant changes in bone density [55,56].

### 4.2. Bone Scan

Bone scans are one of the most popular, highly sensitive diagnostic imaging techniques used for the early detection of the healing process against any bony destruction. In the case of any bony destruction originated due to traumatic, infectious, neoplastic, or benign and malignant diseases of other origin, the lytic area is surrounded by an intense osteoblastic healing process. These significant metabolic changes due to local bone remodeling are detected in a bone scan through a radiotracer that accumulates in the skeleton in proportion to local blood flow. This increased unbound radiotracer uptake is rapidly cleared from the surrounding soft tissues after intravenous injection. A bone scan may detect these significant metabolic changes much earlier than other conventional radiological images. In addition, a bone scan performs a complete skeleton examination covering a wide range of bone disorders at relatively low radiation exposure [57,58]. However, imaging devices are bulky, costlier, and need labs for assessment [59].

### 4.3. Bone X-ray (Radiography)

Bone X-rays have been the primary tool for imaging the stress-related bone injuries. Being relatively cheaper and widely available, they are popularly used in clinical practice worldwide. Radiographic findings are usually “normal” if obtained within the first week of pain; however, in follow-up radiography, the diagnostic findings of bone injuries are present in 30% to 70% of cases [60,61]. Only a limited part of the skeletal system can be visualized through radiography [62,63]. Bone imaging techniques have various advantages and limitations depending upon the need and clinical history of patients [64].

### 4.4. Calcium Blood Test

The amount of calcium in blood is determined by a calcium blood test [65]. Nearly all (99%) of calcium is stored in bone, and the remaining 1% is found in blood. If blood calcium decreases or increases, this may be a sign of bone diseases [66]. Calcium isotope measurement in blood is more sensitive to changes in bone mineral balance (BMB) when compared to existing clinical techniques. Net BMB can inherently be monitored by modeling Ca isotopes; also, Ca isotopes can detect any shift in BMB much earlier (weeks to months earlier) on occurrence when compared to radiological detection of changes in bone mineral density [67]. However, it gives very limited information related to bone health.

### 4.5. Bone Biopsy

The bone biopsy is a technique in which tissue or cells are removed by surgery and the outer layer of bone is analyzed under a microscope [68]. Needle biopsy and open biopsy are two different methods used for taking samples. Bone biopsy causes some complications such as bone fracture, discomfort, bleeding, and infection near the biopsy site [69]. Additionally, it gives information of only the tested area of infection, and it cannot identify the health loss prior to disease.

## 5. Biochemical Markers for Bone Health Monitoring

Biochemical markers of bone turnover are protein derivatives and collagen breakdown products released during the bone remodeling process. Bone turnover markers (BTMs) offer prognostic information of fracture risk and are found in blood and urine. Hannon et al. have reviewed the bone markers and current laboratory assays [70]. Various BTMs are briefly described below.

### 5.1. Biochemical Markers for Bone Formation

Bone biomarkers are produced during bone remodeling and are responsible for bone formation [71]. Bone specific alkaline phosphatase (BALP), procollagen I carboxyterminal propeptides (PINP), procollagen I aminio-terminal propeptide (PICP), and osteocalcin are common biomarkers used for the bone health assessment [72]. The detection of these biomarkers shows potential for the early diagnosis of bone diseases.

#### 5.1.1. Bone-Specific Alkaline Phosphatase (BALP)

Bone-specific alkaline phosphatase (BALP) is generated by osteoblast during the bone remodeling process, and its production is related to bone formation proportion as measured by histomorphometry [72]. BALP levels remain stable in men throughout life; however, the BALP level in women increases around menopause [73].

#### 5.1.2. Procollagen I Peptides

There are basically two types of procollagen peptides, which are described as procollagen I carboxyterminal propeptides (PICP) and procollagen I aminioterminal propeptide (PINP) [74,75]. PINP is stable at room temperature and has low diurnal variability. Its value during circulation is not affected by food intake, and hence there is no need for fasting during tests [76].

#### 5.1.3. Osteocalcin

Osteocalcin is a biomarker produced during the osteoblast activity of bone formation [77]. It is discharged into urine by glomerular filtration [78]. The OC level is low up to middle age in men and increases thereafter; however, in women, it follows the same pattern as in BALP [79].

### 5.2. Biochemical Markers of Bone Resorption

#### 5.2.1. Hydroxyproline (OHP)

Hydroxyproline is an impartial heterocyclic protein amino acid found in collagen [80]. During the resorption process of bone, 90% of OHP is released during collagen breakdown, and the remaining 10% releases as small polypeptide chains [81].

#### 5.2.2. Collagen Cross-Link Molecules

Pyridinoline (PYD) and deoxypyridinoline (DPD) are two types of cross-link molecules that grow during the extracellular maturation of collagen and release into circulation after bone resorption [82]. PYD is found only in the cartilage, ligaments, and vessels of bone. However, DPD is found in bone as well as in dentin [83]. Therefore, DPD is considered a more sensitive marker as compared to PYD.

#### 5.2.3. Hydroxylysine Glycosides

Hydroxylysine glycosides are a type I collagen that are derived from proline and produced by post-translational hydroxylation [84]. This marker is not affected by any food intake and is a more specific bone marker when compared to OHP [85].

#### 5.2.4. Telopeptides of Type I Collagen

These telopeptides are cross-linked and derived from the carboxyterminal (CTx-I) and amino-terminal (NTx-I) during the resorption process of bone. CTx-I and NTx-I are analyzed through immunoassay in urine and serum [86]. The measurement of these two collagens from serum are more sensitive and practical as compared to those from urine [87,88].

#### 5.2.5. Bone Sialoprotein (BSP)

The large amount of BSP is produced during osteoblasts, and a much smaller amount is produced in odontoblasts and osteoclasts. Serum is used for the analysis of BSP [89].

#### 5.2.6. Tartrate-Resistant Acid Phosphatase (TRACP)

TRACP are enzymes found in bones, erythrocytes, platelets, spleen, and prostates released during circulation [90]. TRACP5a and TRACP5b have similar structures but different pH and carbohydrate values. TRACP5a is released from macrophages and TRACP5b is released during osteoclasts, which shows the depravity of the bone matrix. The functioning of kidney and food intake does not affect the level of TRACP5b [91].

#### 5.2.7. Cathepsin K

Cathepsin K belongs to the cysteine proteases group and is released during bone resorption [92]. It plays a vital role in bone matrix degradation. The excessive bone loss treatment is done by cathepsin k inhibitors [93].

## 6. Traditional Techniques for Measurement of Bone Turnover Markers

The bone turnover markers can be analyzed with different methods such as enzyme-linked immunosorbent assay (ELISA), radioimmunoassay (RIA), and high-performance liquid chromatography (HPLC).

### 6.1. Enzyme-Linked Immunosorbent Assay (ELISA)

It is a plate-based assay technique for the detection of antibodies or antigens by color variation in biological fluid. The detection of target molecules depends on the antibody–antigen interaction [94]. The ELISA method is mostly used for the detection of peptide and protein. Enzyme immunoassay can be either homogeneous or heterogeneous. In the homogeneous method, enzymes become inactivated when bound with antibody, and washing is not needed, making it easier to use. However, this method is costly and less sensitive [95]. The heterogeneous method being highly sensitive is more popular comparatively. This technique relies on the formation of an antigen–antibody complex, while free antigens are removed during washing [96].

### 6.2. Radioimmunoassay (RIA)

Radioimmunoassay (RIA) is used for the detection of antigen and antibody. In this method, in the place of enzymes, radioisotopes are used as labels to be conjugated with antibodies or antigens [97]. The sensitivity of RIA is more than that of ELISA [98]. This test is used to measure a very less (nanograms) quantity of antigens and antibodies in serum. RIA works on the viable binding of antibody with unlabeled antigen and radiolabeled antigen [99]. There are some limitations such as costly equipment and reagents, the disposal of radioactive waste, and the short shelf-life of radiolabeled components [100].

### 6.3. High-Performance Liquid Chromatography (HPLC)

The advanced form of column liquid chromatography to separate a combination of substances into their constituents, based on their composition and molecular structure, is used in HPLC [101]. It offers several advantages in terms of the measurement technique of biochemical markers, owing to its high resolution, sensitivity, precision, low response time, and the ability to measure multiple component in single analysis [102].

Detection processes used in ELISA and RIA are highly specific and sensitive. In addition, ELISA requires minimum reagents and has no radiation hazard. HPLC has its own advantages though, as it measures accurately with high speed, sensitivity, and resolution. This automated technique can analyze multiple chemical components in a single analysis [92]. However, in ELISA, technical expertise is needed to prepare complicated samples, and results may also not be absolute, even using expensive ELISA kits. Similarly, RIA also requires technical expertise to make special arrangements for the storage and disposal of radioactive materials and avoid radiation hazards [91]. HPLC has no such limitations; however, it is expensive and uses complex equipment [94].

## 7. Sensors for Diagnosis of Bone Health

Two types of general sensors (physical sensors and biosensors) can be used for the diagnosis of bone health. A physical sensor is a device that measures a physical quantity (such as temperature, strain, pressure et al.) and converts it into a signal that can be read by an observer or by an instrument. A biosensor is an analytical device that is used for the detection of a chemical substance, which includes a biorecognition domain, transducer, and signal read-out system [103,104]. The following section will discuss these two types of sensors for the diagnosis of bone health.

### 7.1. Physical Sensors

**Figure 4:**
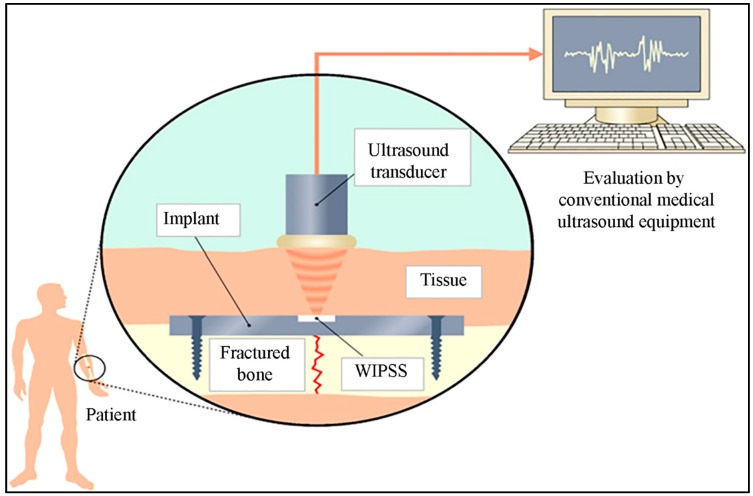
Schematic of the sensor concept of wireless implantable passive strain sensors (WIPSS) implant inside the body and sensor signal is detected by ultrasound waves (reproduced with permission from reference [109], copyright IOP).

Physical sensors monitor and measure force, tension, pressure, weight etc., which can be converted to a measurable signals such as electrical resistance [105]. In addition to these, physical sensors are also sensitive to the surrounding environment and temperature [106]. Thus, they primarily suffer from the limitations of non-specific analyte interactions [107]. Wen et al. shared an implantable strain gauge sensor array fabricated at microscale that can measure surface strain on a live bone. In this study, metal strain gauges encapsulated in a polydimethylsiloxane (PDMS) membrane showed more accurate strain sensing when compared to commercially available ones. This comparison is analyzed by an electromechanical test, which represents accurate strain sensing. This study reflects that the strain gauge may be implantable, and it can wirelessly perform real-time in vivo monitoring during bone remodeling [108]. In 2010, Umbrecht et al. presented wireless implantable passive strain sensors (WIPSS) to observe the disfigurement of orthopedic implants (Figure 4). The WIPSS was made from biocompatible PMMA, and an incompressible fluid was filled in the reservoir. The sensing principle of this sensor was based on amplification effect in hydromechanical systems. The strain resolution obtained was 1.70 ± 0.2 × 10^−5^ with a dynamic input frequency range of 0.1–5 Hz, while this sensor works with a signal bandwidth up to 1 Hz, since increasing the input frequency range reduces the sensor output [109].

In 2009, Lin et al. investigated a smart polymer hydrogel thin film used to convert tiny pressure sensors into chemomechanical sensors. The smart hydrogel was placed between a porous membrane and the diaphragm of a piezoresistive pressure transducer. The sensitivity was affected by the loading pressure and the selection of the membrane. This sensor was used in physiological monitoring [110]. The magnetoelectric (ME) effect represents the coupling of magnetic and electric properties of materials, which can be used in physical sensors to report the signal. In this type, electrical output is produced in response to the magnetic field applied [111,112]. Magnetic sensors are stable, without background noise and high sensitivity [113]. Naughton et al. disclosed in a patent the development of a magnetic biosensor for the monitoring of bone tissue growth. In this invention, they placed a magnet close to bone tissue, whereby the distance between the bone and sensing interface allows the determination of the thickness of bone tissue and in turn, the status of bone tissue degradation [114]. Physical sensors can report the optical signal as well. Singh et al. proposed a model of fiber-optic sensors (FOBs) using the micro bending technique for the measurement of bone strength. They also used an artificial neural network-based test bench for optimization of the FOBs [115]. Optical sensors are faster, having no electrical and magnetic interference with label-free detection. Two drawbacks are its low spectral resolution and bulky system, making its portability poorer [116].

**Figure 5:**
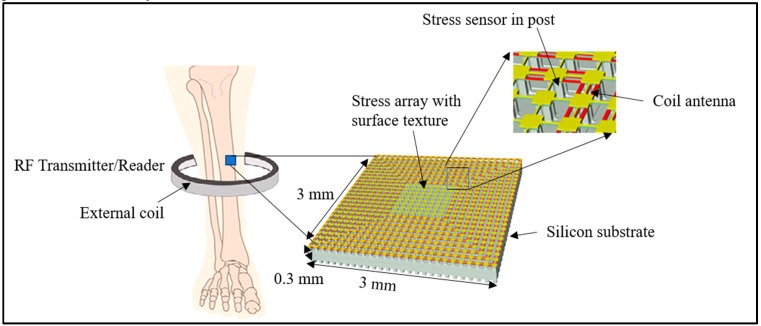
Schematic diagram of an implantable piezoresistive stress sensor (reproduced with permission from reference [13], copyright IOP).

Another type of physical sensors are piezoelectric sensors, which are a group of analytical devices that uses the piezoelectric effect to measure changes in force, strain, temperature, pressure, and acceleration in the form of an electrical charge. Piezoelectric biosensors offer real-time and label-free transduction [117]. Alfaro et al. designed a micro-scale implantable multi-axial bone stress sensor, as shown in Figure 5. As reported, such biosensors work on a piezoresistive pixels array, which can detect a stress between the bone and biosensor chip at an interfacial area with a pressure of around 100 Pa [13]. Piezoelectric sensors show rapid response, easy-to-use, label-free detection, low cost, and negligible phase shift with a compact size and offer output that can be directly processed by an electronic circuit. Piezoelectric sensors are also sensitive to temperature and pressure, which may also become a drawback [118].

The above-discussed signal (physical, magnetoelectric, piezoelectric, and optical) read-out methods used in physical sensors can also be used for biosensors [119,120,121]. However, so far, no biosensor using these types of signal read-out has been used for the detection of bone biomarkers to diagnose bone health.

### 7.2. Biosensors

In recent years, the requirement of biosensors has increased due to its fast response time, user-friendly approach, minimum cost, disposable device, and suitability for mass production. Biosensors can be very useful in bone health monitoring to timely and continuously assess issues such as fracture, the reduction of BMD, and variation in a variety of proteins [122]. Various technologies are evolving in the field of biosensors that can assess bone cells and identify the concentration of BTM in biological samples. In general, bone biosensing comprises a recognition domain that identifies the analyte that gets converted into a signal through a signal transducer, and a reader device reads this signal [123]. Advances in biosensing technology have enabled producing reliable, fast, non-invasive, real-time, and cost-effective sensors with high sensitivity that can help with precisely monitoring the bone health [124]. A bone biosensor fabrication process involves three different stages: (1) the selection of transducers; (2) fabrication of a sensing interface with recognition elements; and (3) quantitative measurements through the signal amplification and transduction element [125]. Modification of the sensing interface with nanomaterials is the attractive practice for the fabrication of a biosensor. Nanomaterials such as gold nanoparticles, carbon dots, nanorods, nanotubes, quantum dots, and nanowires-based biosensors have shown great potential in diagnostics, owing to their unique properties such as high electrical conductivity and large surface area, resulting in high sensitivity [126,127]. Based on signal read-out strategies, there are different types of biosensors such as colorimetric biosensors, fluorescence biosensors, electrochemical biosensors, et al. The following section will discuss the current biosensors that have been used for bone health studies.

#### 7.2.1. Electrochemical Biosensors

**Figure 6:**
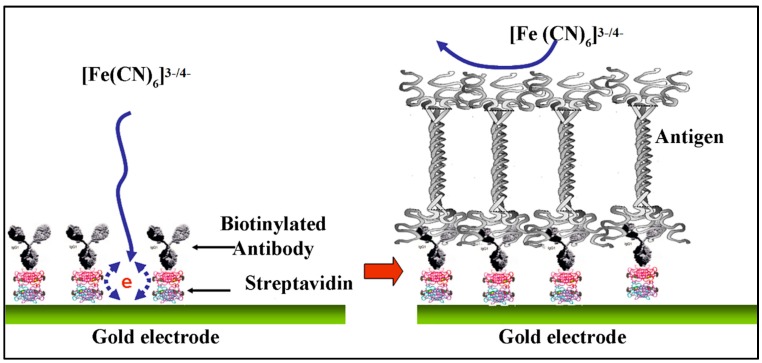
Schematic diagram of label-free immunobiosensor based on the detection of carboxyterminal (CTx-I, reproduced with permission from reference [132]).

Electrochemical biosensors can convert biological information into a measurable electrical signal using simple electronics for conditioning and read-out [128]. These biosensors are advantageous in terms of linear output, excellent repeatability, reliable, portable, accurate, and require less power [129]. On the other hand, the shelf-life, stability of biorecognition element, non-specific binding, and ultra-sensitivity to temperature are the limitations of electrochemical biosensors [130]. Ramanathan et al. developed immunosensors for the impedimetric detection of bone biomarkers (CTx-I) that can detect with a low limit (0.05 ng/mL) of detection [131]. Yun et al. developed a label-free immunosensor for detection of the C-terminal telopeptide bone turnover marker from type-1 collagen [132]. In this work, self-assembled monolayers of dithiodipropionic acid were used on the surface of gold electrodes with streptavidin immobilized, following which a biotinylated antibody was bound to the streptavidin. The different concentrations of CTx-I were measured through electrochemical impedance spectroscopy (EIS) (Figure 6). A detection limit of 50 ng/mL and a dynamic range up to 3 μg/mL was achieved. It was reported that using this method, the sensors can measure the electrical signal in just 4 h through a single step as opposed to commercially available methods such as ELISA, which takes a lot more time and a greater number of steps for analysis.

**Figure 7:**
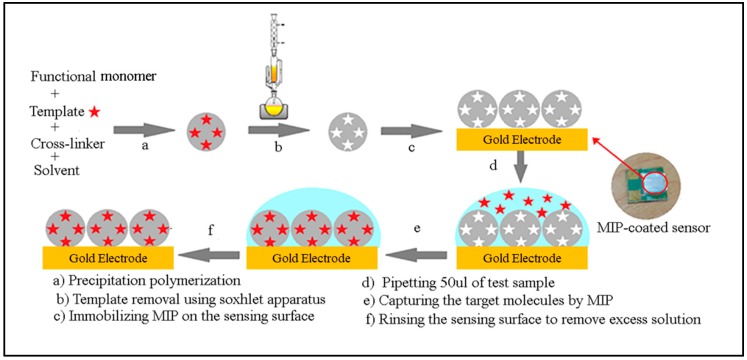
Process flow scheme for the preparation of molecularly imprinted polymer for CTx-I recognition (reproduced with permission from reference [134], copyright 2018 IEEE).

In 2016, Afsarimanesh et al. reported another label-free biosensing technique by monitoring the CTx-I concentration in serum [133]. The impedance variation of CTx-I at different levels was analyzed by a combination of EIS and interdigital sensor. A detection limit of 0.147 ng/mL was attained through this method, and the results were compared with ELISA for validation. In 2018, the same research team represented a new method for CTx-I detection in serum [134]. Artificial antibodies were prepared for CTx-I molecules by the molecular imprinting (MIP) technique, as shown in Figure 7. Dielectric properties of the test solution were analyzed by a combination of EIS and an interdigital capacitive sensor. The detection limit was reduced up to 0.09 ng/mL, and the results were validated using ELISA. The authors reported that this biosensor performed better as compared to ELISA.

In 2019, Inal et al. developed a biosensor for the prognostic monitoring of osteoporosis by measuring osteocalcin molecules [135]. An anti-osteocalcin antibody was immobilized via the covalent immobilization method onto a gold electrode surface. Biosensor characterization and immobilization were specified by cyclic voltammetry and electrochemical impedance spectroscopy. The osteocalcin concentration was detected in 45 min with a detection range of 10–60 pg µL^−1^. Sappia et al. developed an electrochemical biosensor, which they reported to be efficient, promising, and a simple technique for alkaline phosphatase (ALP) determination. It showed potential to be used by physicians for clinical tests requiring only 10 µL of serum even through a finger prick [136]. In 2009, Chandra et al. developed multiplexed assay for rheumatoid arthritis (RA) with more sensitivity and specificity than clinical tests [137].

#### 7.2.2. Acoustic Biosensors

Acoustic biosensors are non-invasive in nature [138]. The emission parameters and accumulation number of recorded activities are correlated to determine the magnitude of bone damage [139]. Lentle et al. disclosed in a patent the use of acoustic biosensors to detect the osteoporosis level through acoustic sound waves by placing the sensor with skin adjacent to bone. In this invention, applied acoustic emission is detected by a biosensor, which in turn analyzes the sound waves to evaluate the extent of osteoporosis in bone [140]. Acoustic biosensors are cheaper, sensitive, provide multiplexed output, wirelessly interrogated, and able to work in liquid environment with low detection limit [141,142]. The main disadvantages of acoustic sensors are their dependence on temperature and humidity, which makes sensor replacement difficult, and the sensor sensitivity also depends on thin crystal [143].

#### 7.2.3. Other Sensors

**Figure 8:**
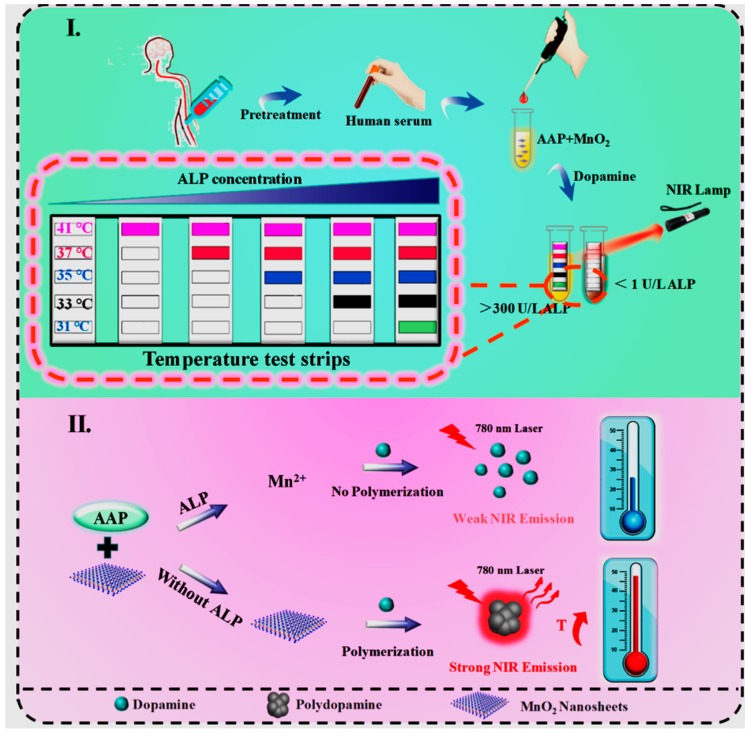
Photothermal biosensor for the measurement of ALP (reproduced with permission from reference [144], copyright 2019 American Chemical Society).

In addition to the popular electrochemical and acoustic biosensors, recently Liu et al. developed a portable photothermal biosensor to detect ALP enzymes on the basis of polydopamine (PDA) nanoparticle formation by using a thermometer as a read-out or temperature discoloration sticker, as indicated in Figure 8 [144]. This biosensor can work on a very low detection limit of 0.1 unit/length (U/L) for a thermometer and 1.0 U/L for a temperature discoloration sticker. As suggested, this type of biosensor can detect ALP in serum with very high sensitivity and label-free detection in real time.

Although the aforementioned examples are not exhaustive, they have demonstrated the huge potential to produce reliable, fast, non-invasive, real-time, and cost-effective biosensors with high sensitivity that can help in efficiently monitoring the bone health.

## 8. Conclusions and Future Trends

This paper focuses on different methods for the assessment of bone health using biochemical markers. Modeling and remodeling phenomena of bones, various bone diseases, and bone health monitoring techniques are briefly discussed. The paper highlights a variety of bone turnover markers and recent developments in this area of monitoring biochemical markers for bone health assessment. Then, the paper discusses recent bone biosensing techniques and types of biosensors, with an emphasis on electrochemical biosensors for bone health monitoring, owing to the high sensitivity and reliability.

However, it is important to note that the available sensors have certain limitations and challenges for widespread applications. Therefore, there is a critical need to further explore and develop biosensors for real-time monitoring with simpler, quicker, more cost-effective and user-friendly approaches. The majority of bone biosensors are used to detect a single biomarker of bone health. Single bone biomarker detection is not sufficient for the accurate and timely identification of bone diseases, and hence, the detection of multiple biomarkers is required to be developed. Therefore, methods for multiplex detection, which can detect multiple analytes simultaneously, are required to overcome these problems. It has been reported that multiplex assays have high sensitivity and require less sample volume. The development of advanced biosensors that may detect multiple markers with label-free detection technique are needed to solve the ever-increasing bone health problems in the near future.
